# Case Report: Diagnosis of multiple juvenile angiomatosis in a newborn calf

**DOI:** 10.3389/fvets.2026.1882023

**Published:** 2026-08-17

**Authors:** Takeshi Tsuka, Kodai Kusuda, Sohta Hishikawa, Natsuki Hashimoto, Yuji Sunden

**Affiliations:** 1 Department of Veterinary Clinical Medicine, Joint Department of Veterinary Medicine, Faculty of Agriculture, Tottori University, Tottori, Japan; 2 Kusunoki Large Animal Clinic, Tottori, Japan

**Keywords:** computed tomography, core-needle biopsy, fine-needle aspiration, histopathology, ultrasonography

## Abstract

Juvenile angiomatosis is a rare vascular anomaly that has never been examined by ultrasonography and computed tomography in bovine practice. A four-days-old, Japanese-Black, male calf presented with multiple swollen skin lesions aligned along the dorsoventral axis of the left thoracic wall. Ultrasonography showed homogeneous echogenic masses between the subcutaneous and muscular structures. Anatomical connection between the mass lesions and neighboring structures was ultrasonographically unclear based on differences in echogenicity and the hyperechoic membranous structures between them. Doppler ultrasonography revealed poor vascularity, which is a common feature of mass lesions. Computed tomography (CT) revealed seven masses in the left thoracic wall. Five of the seven masses were isolated from each other, although there was an anatomical connection between two masses formed at the level of the spinous processes of the ninth to thirteenth thoracic vertebrae. The largest mass was located in the most ventral region of the thoracic wall, corresponding to deformities of the sixth to tenth ribs, which showed severe inward rolling into the thoracic cavity on three-dimensional CT. Core-needle biopsy allowed histopathological observation of the predominant intra-mass configuration of vessel-like structures. Histopathological examination of tissue samples collected at necropsy revealed that multiple immature vessel-like structures were present within the mass lesions that commonly involved the skeletal muscles. Positive immunostaining was found for von Willebrand factor in the endothelial cells, and for alpha-smooth muscle actin in the cells comprising the walls. These findings could support a diagnosis of bovine juvenile angiomatosis. Both ultrasonography and CT provided no definitive evidence to support the macroscopic and histopathological findings showing invasion of the masses into neighboring muscular structures. A combination of diagnostic techniques, including ultrasonography, CT, and core-needle biopsy, helped provide complementary information that supported the histopathological and immunohistochemical diagnosis of this disease.

## Introduction

Angiomatosis is a benign vascular anomaly whose pathogenesis is not fully understood (1–3). Based on the ISSVA (International Society for the Study of Vascular Anomalies) classification of vascular anomalies (2025), this disease is categorized as a vascular tumor rather than a vascular malformation (4). In veterinary medicine, this condition and vascular hamartoma are often regarded as the same vascular malformation entity (5). One previous bovine report defined angiomatosis as a hemangioma-like disease (6). In human medicine, angiomatosis is defined as multiple hemangiomas and is used synonymously with diffuse hemangioma (7). Hemangioma types, categorized in human medicine as congenital or infantile, should be clearly distinguished from vascular malformations because they belong to different pathological categories (2, 8, 9). However, the term hemangioma is often misused because these lesions can be difficult to distinguish as either tumors or malformations (2). Congenital hemangiosarcoma also cannot be completely differentiated from angiomatosis, vascular hamartoma, and hemangioma, although it is categorized as a malignant tumor (10). To reduce confusion in the classification of these diseases, which have been described by various names (11, 12), the term bovine juvenile angiomatosis is proposed to encompass both congenital vascular tumors and vascular malformations (1, 9, 13–15). On the other hand, a hereditary basis has been considered for bovine juvenile angiomatosis as well as hemangioma (1, 6, 15, 16). The term bovine juvenile angiomatosis is also useful for distinguishing this condition from bovine cutaneous angiomatosis, which occurs mainly in adult cattle and is thought to arise from either an abnormal reactive response or an idiopathic hamartoma (15, 17). Similar acquired mechanisms of cutaneous angiomatosis are common in human patients (3, 8).

Angiomatosis has been detected in multiple organs, including the heart, liver, spleen, kidneys, and spinal canal, as well as in the skin, in previous bovine cases, although the skin was the predominant site of involvement (1). Cutaneous and subcutaneous involvement, which occurs predominantly in bovine cases, can be readily detected because of swelling in the affected regions (1). However, swollen cutaneous surfaces are common gross findings in various superficially located mass lesions, including spina bifida cystica, cellulitis, abscesses, tumors, and tumor-like lesions such as hemangioma, hamartoma, and choristoma (18, 19). In particular, when diagnosing angiomatosis, differentiation from hemangioma and other proliferative vascular lesions is required (3). Thus, diagnostic techniques are needed to objectively distinguish this disease from other vascular diseases.

Angiomatosis can be differentiated from these mass lesions using imaging modalities such as radiography, ultrasonography, and computed tomography (CT) in both human medicine (2, 14, 20–25) and veterinary practice (3, 19, 26–34). Ultrasonography is clinically more informative than radiography, because radiography provides limited resolution of soft tissue structures (21). However, some veterinary reports have described the utility of ultrasonography in diagnosing angiomatosis (3, 30, 31). CT has been used effectively to evaluate vascular anomalies (19, 27–29). It can identify abnormalities in the primary lesion and in adjacent secondary changes involving soft tissues and bone within a single examination (19, 28, 29). Several veterinary and human case reports are available on the clinical utility of CT for evaluating angiomatosis (27, 28, 31, 35–37).

The present report provides a comprehensive antemortem evaluation of ultrasonography and CT, followed by postmortem diagnosis of juvenile angiomatosis through necropsy and histopathological examination. The present findings are discussed in comparison with previous human and animal reports, with a particular focus on the clinical contribution and limitation of these two imaging modalities in diagnosing this disease.

## Case presentation

**Figure 1:**
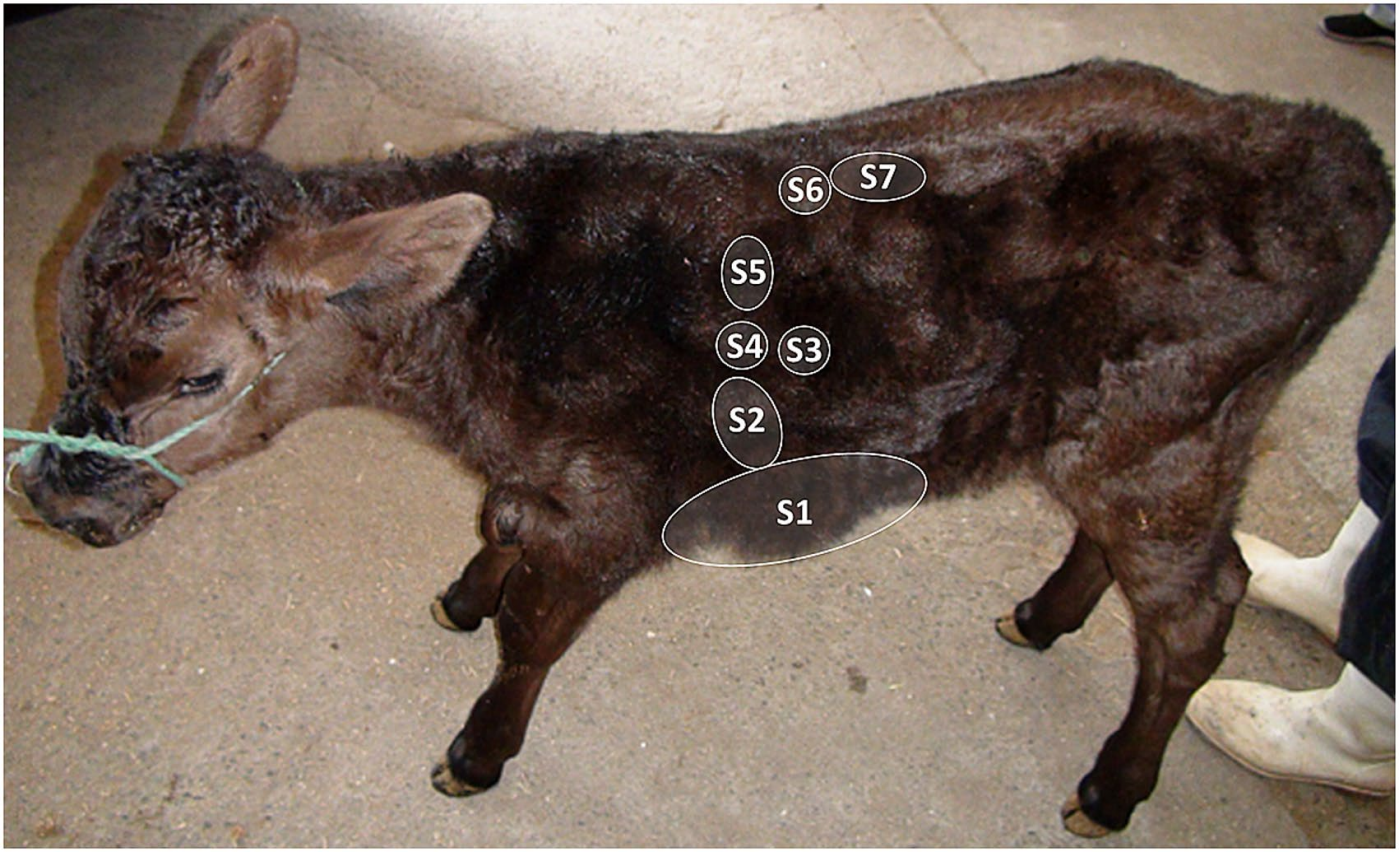
Photograph of the left thoracic wall in a Japanese Black male newborn calf. Swelling is evident in seven regions (S1 to S7) aligned along the dorsoventral axis of the left thoracic wall.

A four-days-old, Japanese Black male calf presented with swelling extending from the dorsal to ventral aspects of the left thoracic wall since birth (Figure 1). The calf was in poor physical condition, showing growth retardation and a gradual decrease in body weight (35 kg), despite its normal suckling of the dam’s milk. Palpation revealed at least seven swollen areas aligned dorsally to ventrally along the left thoracic wall. The largest mass, measuring 20 cm in length and 10 cm in thickness, referred to as S1 in the present study, was located in the most ventral region of the left thoracic wall, extending along the craniocaudal axis of the body. The S1 mass was palpated as a large parenchymal structure without a fluid-filled cavity at the level of the sternum and along the fifth to eleventh ribs. The S1 mass could not be pushed into the body cavity. The second mass (S2) was palpated as a 5 cm long oval parenchymal structure located dorsal to the middle area of the S1 mass, and at the level of the fifth to seventh ribs. The third and fourth masses (S3 and S4, respectively) were aligned at the cranial and caudal aspects of the thoracic wall dorsal to the S2 mass. The S3 and S4 masses were located at the level of the sixth to seventh ribs, and the seventh to ninth ribs, respectively. The fifth mass (S5) was located dorsal to the S4 mass at the level of the sixth to ninth ribs. The sixth and seventh masses (S6 and S7, respectively) were palpated as firm cranial and caudal masses aligned at the level of the ninth to thirteenth thoracic vertebrae. None of the masses were movable on palpation, suggesting either fixation to or origin from the surrounding soft tissue structures. In addition, no anatomical connections were observed between the masses and the overlying skin.

**Figure 2:**
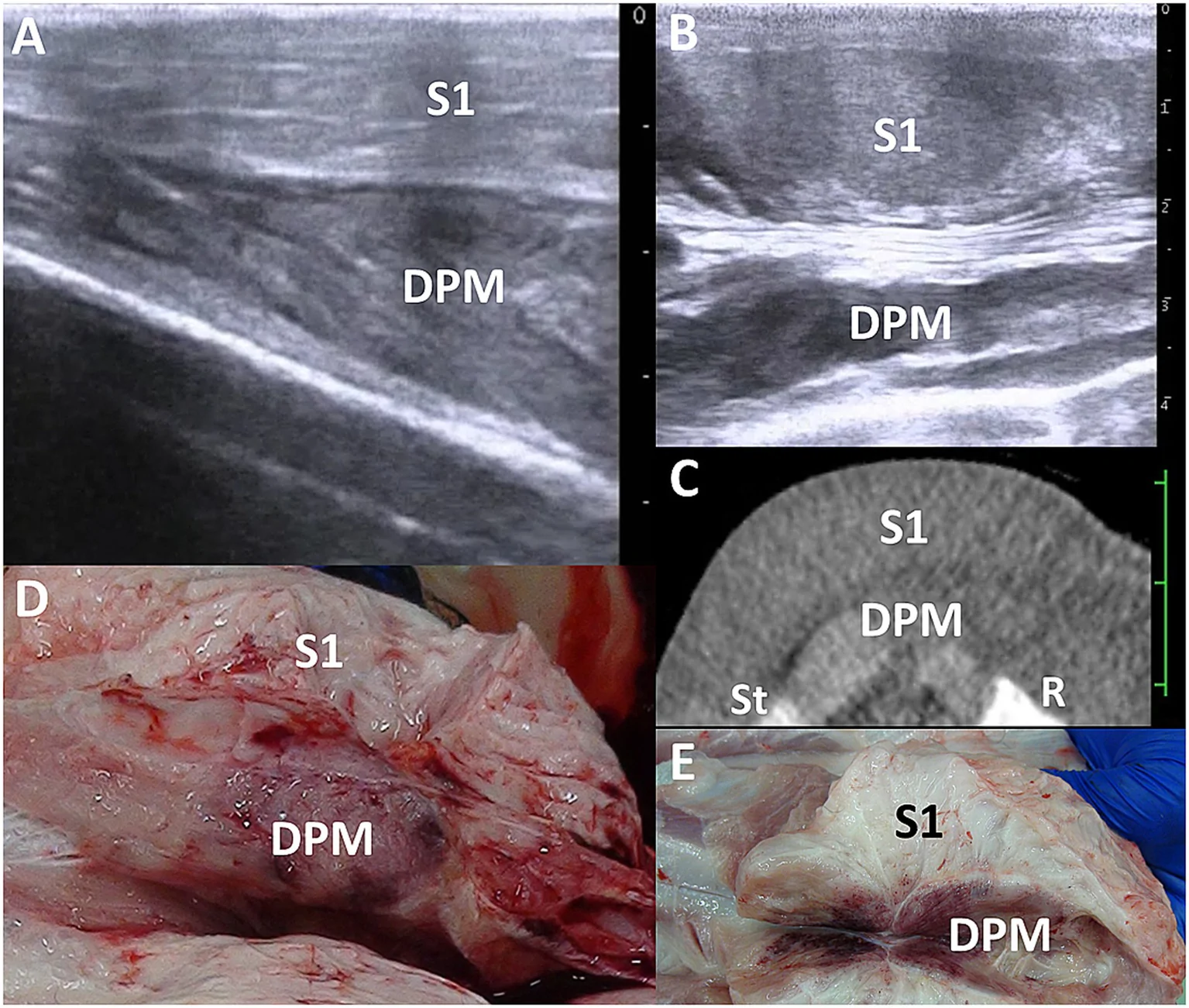
Longitudinal and transverse ultrasonograms (**A,B**, respectively) transverse computed tomographic image **(C)** and sagittal and transverse macroscopic views of the S1 mass (**D,E**, respectively). **(A)** The S1 mass runs along the underlying layer of the deep pectoral muscle (DPM), and the border between them is distinct because of the difference in echogenicity. **(B)** The echogenic S1 mass and the DPM, which shows mixed echogenicity, are seen superficially and deeply, respectively. Tendinous structure runs between the S1 mass and DPM. **(C)** Thickening is evident in the S1 mass, which is located superficial to the DPM extending between the sternum (St) and the left rib (R). **(D)** Well-defined layers are present between the S1 mass, containing adipose tissue, and the discolored DPM. **(E)** The S1 mass, containing adipose tissue, runs adjacent to the discolored DPM. Scale bars: 10 mm on ultrasonography and 25 mm on computed tomography.

**Figure 3:**
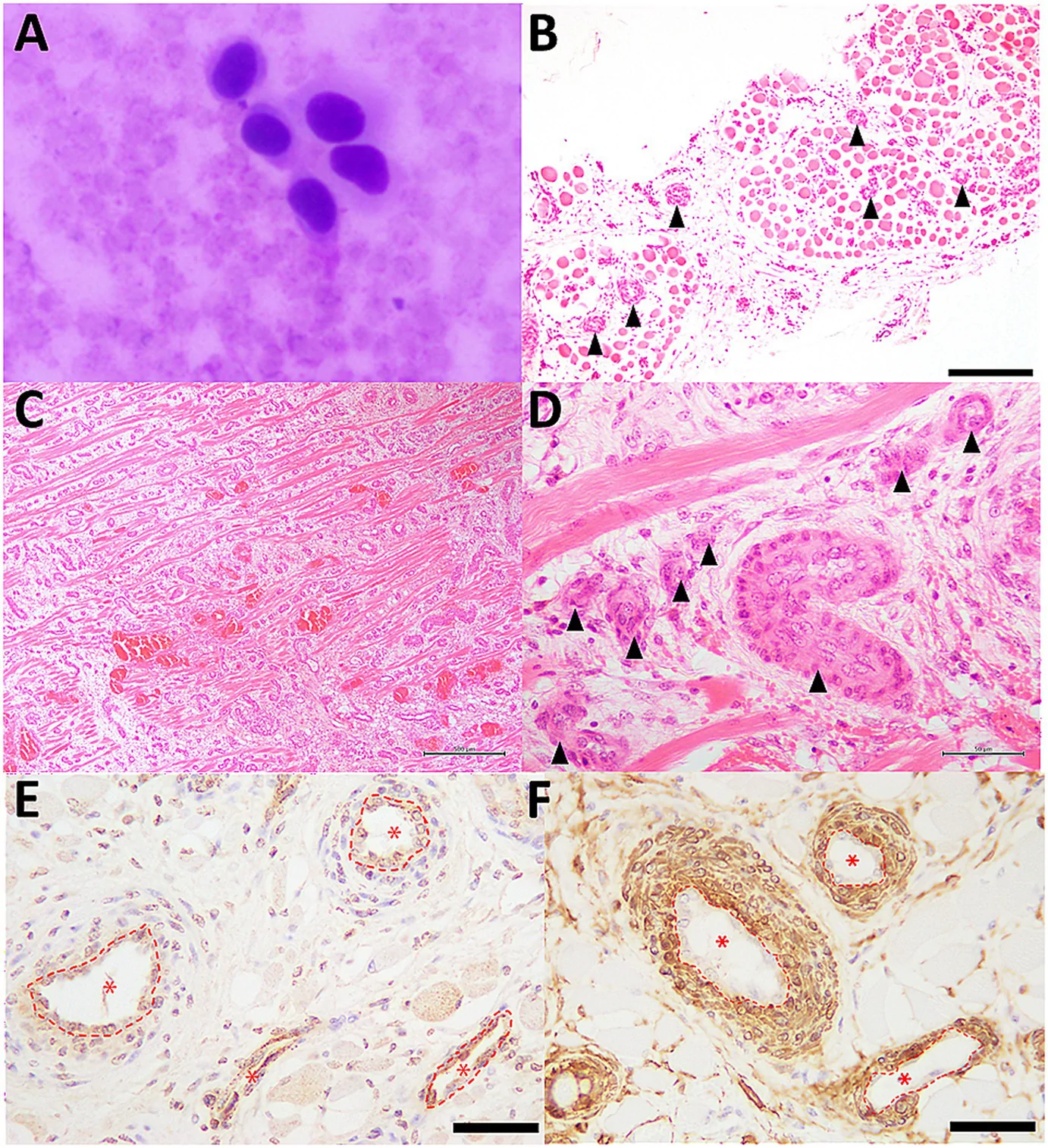
Cytological view of the fine-needle aspiration specimen **(A)**, histopathological sections of the core-needle biopsy specimen **(B)** and the specimen collected at necropsy **(C,D)**, and immunohistochemical sections of the specimen collected at necropsy **(E,F)**. **(A)** Large cells with intensely stained nuclei are present. **(B)** Vessel-like structures lined by thickened walls (arrowheads) are scattered among slightly atrophic muscle fibers. Hematoxylin and Eosin (H&E) staining. Bar = 200 μm. **(C)** Vessel-like structures containing erythrocytes are present between the muscle layers. H&E staining. Bar = 500 μm. **(D)** Thick vessel-like structures composed of concentrically arranged cells are present (arrowheads). H&E staining. Bar = 500 μm. **(E)** The endothelial cells of the structures are positively immunolabeled (brownish signals) with von Willebrand factor. **(F)** The cells comprising the vascular walls are labeled with alpha-smooth muscle actin. Asterisks show vascular lumens and dashed lines indicates the boundary of the endothelial cells. Bars = 50 μm **(E,F)**.

The present case was examined by ultrasonography using a portable ultrasound device (MyLabOne VET, Esaote Corporation, Genova, Italy) in the standing position without anesthesia. A 10 MHz linear transducer was applied to the skin swellings where the S1 to S7 masses could be palpated, while an ultrasound gel was applied to the non-shaved skin overlying the palpable masses. The S1 mass appeared as a homogeneously hypoechoic structure with some hyperechoic striations, outlined by hyperechoic lines, on longitudinal scanning of the most ventral area of the left cranial thorax (Figure 2A). This mass extended along the underlying deep pectoral muscle, which showed disrupted alignment of the muscle fibers. The echogenicity of the S1 mass was higher than that of the underlying muscular structures, allowing a clear distinction between them. On transverse ultrasonography, the S1 mass appeared as an echogenic structure containing scattered hyperechoic spots within the space between the hypoechoic subcutaneous tissues and the hyperechoic tendon-like structures (Figure 2B). Deep to these well-defined layered structures, the deep pectoral muscle appeared heterogeneous, with a mixture of hypoechoic and hyperechoic areas. In the left thoracic wall, the width of the deep pectoral muscle ranged from 1.5 to 2.0 cm, which was greater than that of the approximately 5 mm thick hypoechoic oblong bundles of the same muscle in the normal right thoracic wall (Supplementary Figure S1). The S2 and S3 masses were similarly characterized by spindle-shaped echogenic structures outlined by thin hyperechoic membranous structures (Supplementary Figure S2). These two masses contained segments of hyperechoic structures from the underlying external abdominal oblique muscles, whose echotexture showed normal fiber striations within the hypoechoic structures. Doppler ultrasonography revealed poor vascularity within the S1, S2, and S3 masses, when switching from B mode to color Doppler mode while these masses were imaged in center of the window. The S4, S5, S6, and S7 masses showed echotextures identical to those of the S1, S2, and S3 masses, appearing as homogeneous echogenic structures with an unclear connection to the underlying muscular structures. These four masses were not scanned by Doppler ultrasonography. No anatomical connections among the five masses (S1 to S5) could be identified ultrasonographically, although the connection between the S6 and S7 masses was evident (Supplementary Figure S2). Samples from the S1 mass were obtained by fine-needle aspiration under ultrasonographic guidance without anesthesia. Cytological examination of the specimen revealed an accumulation of large cells with intensely stained nuclei and scant to moderate amounts of cytoplasm, mixed with erythrocytes in the background (Figure 3A). The cytological findings were not specific for assessing malignancy and the derivation of epithelial and mesenchymal lineages.

**Figure 4:**
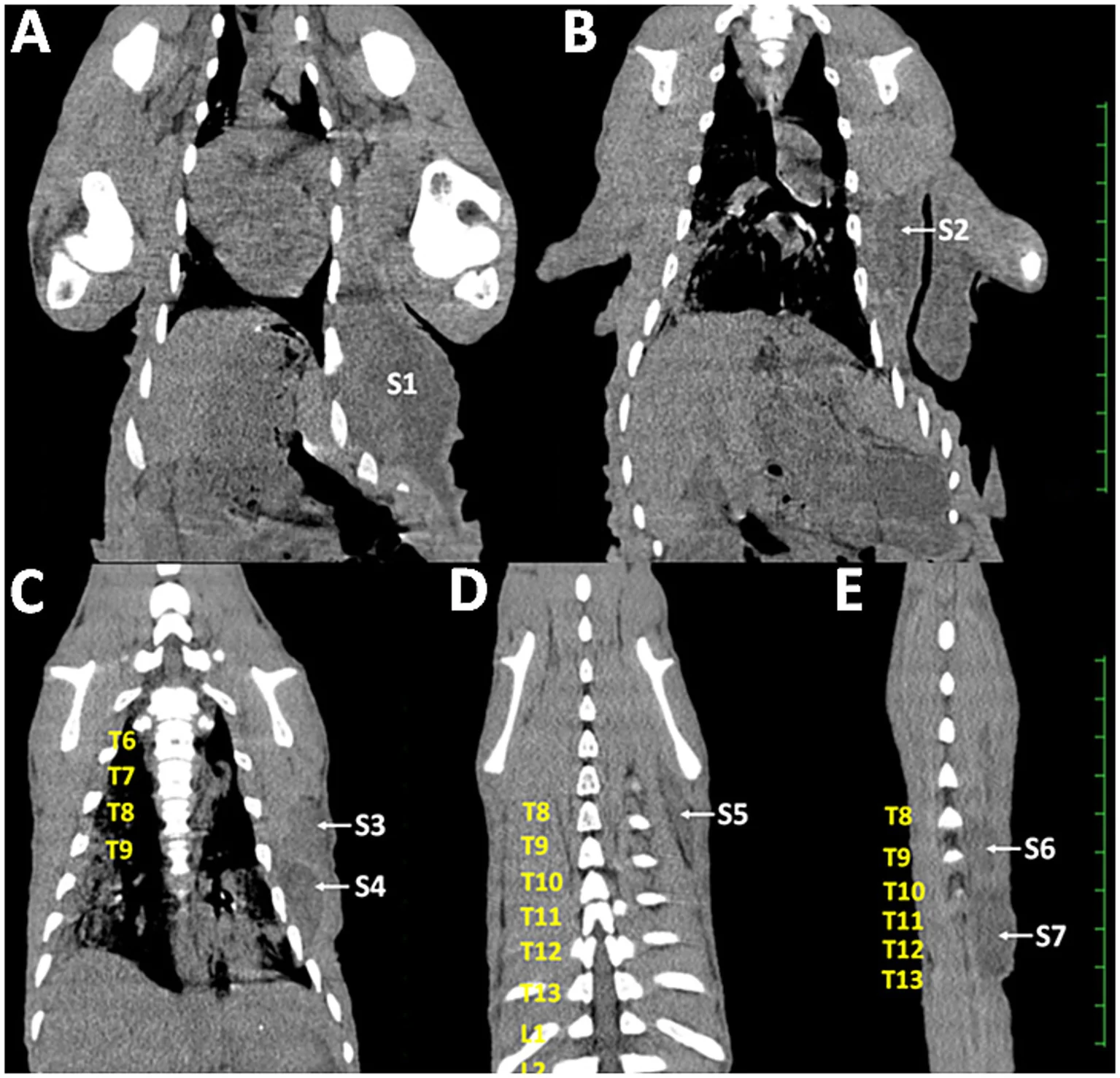
Dorsal computed tomographic sections reconstructed at the most ventral aspect **(A)**, ventral one-fourth **(B)**, middle **(C)**, dorsal one-fourth **(D)**, and most dorsal aspect **(E)** of the dorsoventral body height. **(A)** The location of the largest mass (S1) corresponds to the deformed rib cage. **(B)** The second mass (S2) extends between the sixth and ninth ribs. **(C)** The third and fourth masses (S3 and S4, respectively) are aligned between the eighth and twelfth ribs. **(D)** The fifth mass (S5) appears as a spindle-shaped structure. **(E)** The sixth and seventh masses (S6 and S7, respectively) are present along the left side of the spinous processes of the ninth to thirteenth thoracic vertebrae (T9 to T13). T6 to T13: sixth to thirteenth thoracic vertebrae; L1: first lumbar vertebra; L2: second lumbar vertebra. Scale bar: 25 mm on computed tomography.

The present case was examined using a 16-section multidetector scanner (ECLOS; Hitachi, Tokyo, Japan) with X-ray tube settings of 120 kVp, a current of 175 mA, and a 0.625-mm-slice thickness, while the calf was sedated by intravenous injection of xylazine hydrochloride (0.1 mg/kg), followed by general anesthesia with inhalation of 2%–3% isoflurane via a tracheal tube. On transverse CT of the left ventral thoracic wall at the level of the twelfth rib, corresponding to the ultrasonographic section (Figure 2B), there was a 25-mm-thick S1 mass with soft tissue density extending from the level of the sternum to the middle area of the left rib (Figure 2C). CT revealed different soft tissue densities between the S1 mass and the underlying layers of the deep pectoral muscles, which clearly delineated the border between these structures. The left-sided deep pectoral muscles beneath the S1 mass were thickened compared with the normal right-sided muscle layers, measuring 30 mm and 10 mm, respectively (Supplementary Figure S1). On the dorsal reconstructed CT section showing the ventral aspect of the body, the S1 mass appeared more hypoattenuating than the surrounding muscular structures (Figure 4A). The size of this mass was 172.5 mm in craniocaudal length and 71.7 mm in lateromedial width; the dimensions of the other masses are shown in the same order. This S1 mass showed tail-like extensions at its cranial and caudal edges. Double-tail-like hypoattenuating structures were common CT features in all seven masses. A dorsal CT section at the level of the ventral one-fourth of the dorsoventral body height revealed the S2 mass, measuring 69.0 mm and 34.7 mm, respectively (Figure 4B). On dorsal CT at the middle body height, the S3 mass, measuring 44.5 mm and 30.8 mm, was seen more cranially and superficially than the S4 mass, measuring 54.0 mm and 24.2 mm (Figure 4C). These two masses were located in parallel between the sixth and ninth ribs. The S5 mass was demonstrated as an elongated oval structure measuring 72.6 mm and 18.0 mm between the sixth and ninth ribs at the level of the dorsal one-fourth of the body height (Figure 4D). Dorsal CT at the level of the thoracic vertebrae showed two masses, the cranial S6 mass measuring 36.0 mm by 19.4 mm and the caudal S7 mass measuring 57.7 mm by 27.3 mm, both located between the ninth and thirteenth ribs (Figure 4E). Abnormal curvature of the left ribs toward the thoracic cavity was evident in the region between the sixth and tenth ribs, corresponding to the affected region of the S1 and S2 masses (Figures 4A,B, Supplementary Video S1). The left rib deformities resulted in narrowing of the left thoracic cavity, where parts of the left cranial and caudal lung lobes appeared hypoattenuating. Three-dimensional CT of the rib cage revealed deformities of the sixth to tenth ribs (Supplementary Video S2). The seventh and eighth ribs showed more severe deformities, with their bodies curving to run almost vertically from the dorsal one-fifth portion.

Soon after CT examination under the same anesthesia, tissue samples were collected from the S1 mass by ultrasonography-guided core-needle biopsy using a Tru-cut biopsy needle (Tru-Core™ II Automatic Biopsy Instrument, Argon Medical Devices Inc., Texas, USA). Histopathological examination revealed mild atrophic changes in the muscle fibers (Figure 3B). Scattered vessel-like structures composed of enlarged endothelial cells and thick walls were observed within the muscle tissue.

The clinical examination results indicated a poor prognosis in this case, consistent with the calf’s poor physical condition. Three days after the examinations, the calf was euthanized by intravenous injection of potassium chloride (approximately 2 mmol/kg) under deep anesthesia induced by intravenous injections of xylazine hydrochloride (2 mg/kg) and propofol (10 mg/kg), according to the AVMA Guide 2013.

At necropsy, discoloration of the skeletal muscle, admixed with the mass lesions, was noted especially in S1 mass. On sagittal section of the swollen ventral region of the left thoracic wall, including the S1 mass, the base of this mass was adherent to the underlying deep pectoral muscle (Figure 2D). In addition, most of this mass was embedded within the adipose tissue. A mixture of hemorrhagic and necrotic foci was present within the thickened portions of these muscles. Macroscopic abnormalities extended between two bundles of the deep pectoral muscle beneath the S1 mass, as seen on the transverse section of the ventral thoracic wall between the rib and sternum (Figure 2E). All masses were considered to involve the underlying peripheral skeletal muscles, because the anatomical connections were detected macroscopically (Supplementary Figure S2). In addition, the S1 to S5 masses were anatomically isolated from one another, with no anastomosis between them. On gross examination of the rib cage, specifically between the ninth and thirteenth thoracic vertebrae after removal of the thoracoabdominal organs, the ventral half of the left ribs were sharply curved inward compared with the normal curvature of the right ribs. The abnormal curvature of the left ribs resulted in narrowing of the thoracic and cranial abdominal cavities. No abnormal macroscopic findings were evident in the thoracoabdominal organs. Regarding the other gross findings, four nodules measuring up to 1.2 cm were identified at the base of the mitral valve of the heart. Swelling was present in the internal iliac and superficial lymph nodes, measuring up to 1.2 cm and 3.7 cm, respectively.

Histopathological examination of the masses collected at necropsy revealed that the lesions commonly involved the skeletal muscle. Infiltration of the adipose tissue was also observed, along with fibrous tissue proliferation within the masses. The border between the masses and the muscular structures was poorly defined. Multiple immature vessel-like structures were observed within the intramuscular spaces (Figure 3C). These vessel-like structures were lined with endothelial cells with large, clear nuclei. Their histopathological features varied, including luminal structures lined by one or two layers of flattened endothelial cells and muscle- and artery-like structures with thickened walls composed of several cellular layers (Figure 3D). The lumens of the vessel-like structures varied in diameter, and many contained erythrocytes. Degenerated cells were also observed, including cells with pyknotic nuclei and eosinophilic cells. Hemorrhagic foci and blood clots were present at sites of rupture in the vessel-like structures. A total of six mitotic figures were observed in 20 randomly-selected cross-sections, including vessel-like structures, resulting in an average of 0.3 mitotic figures per cross-section. Mild diffuse infiltration of inflammatory cells, including eosinophils, lymphocytes, and plasma cells, was also present. Mild atrophic changes were focally observed in the skeletal muscle adjacent to the masses. Immunohistochemistry revealed that the endothelial cells were positive for von Willebrand factor, whereas the cells comprising the walls were positive for alpha-smooth muscle actin (Figures 3E,F). Based on these histopathological findings, juvenile angiomatosis was diagnosed in this case.

## Discussion

Vascular tumors and malformations have previously been found at birth or at a young age (1, 6, 10, 11, 13, 15, 16, 27, 30–34). Thus, when diagnosing juvenile angiomatosis, differentiation from these diseases is required (3).

Multiple cutaneous swelling in the affected regions may be a macroscopic finding suggestive of juvenile angiomatosis. This is supported by the course of disease described in a previous bovine case, in which masses were macroscopically detected in the neck, chest, scrotum, knee, and hock after the owner first noticed a facial mass (1). In addition, many previous animal case reports have described multiple cutaneous angiomatosis lesions with diffuse extension across the affected skin surfaces in dogs, cats, horses, sheep, and llamas (3, 5, 7, 8, 12, 26, 28, 30). However, these macroscopic criteria are not always sufficient for diagnosing this disease. Multiple cutaneous lesions can also often be found in human infants with congenital hemangioma, a condition often associated with fatal progression and referred to as diffuse neonatal hemangiomatosis (22). Masses associated with congenital hemangioma and hemangiosarcoma are also frequently multiple in affected bovine cases and have previously been found in skeletal muscle, skin, brain, bone, heart, lung, and various abdominal viscera (6, 10, 16, 38).

Regarding the clinical utility of ultrasonography for diagnosing angiomatosis, only a few reports have been available in human medicine (25, 35, 36) and veterinary practice (3, 30, 31). There were no apparent common ultrasonographic characteristics specific to angiomatosis, based on the previous findings including a solid mass enveloping multiple anechoic cystic lumens separated by thin septa, and a capsulated mass containing anechoic luminal fluids (25, 31). Muscular angiomatosis, resembled with the present case, showed no skeletal abnormality on the ultrasonogram although severe subcutaneous thickening was evident (30). The ultrasonographic findings in the present case resembled those of a lipoma, which appeared as a homogeneous mass with variable echogenicity (14). This result may depend on the histopathological characteristics of the masses in the present case, in which adipose tissue infiltrated extensively within the masses. Thus, ultrasonography appeared to have a limited role in differentiating angiomatosis from other vascular anomalies. In the previous bovine case of juvenile angiomatosis, a blood-filled capsular mass was observed resembling lesions reported in congenital hemangioma and hemangiosarcoma, in which solid or cystic masses containing clotted blood were identified (6, 10, 16, 38). These lesion types may be represented ultrasonographically as heterogeneous hyperechoic structures, based on the typical ultrasonographic findings of hemangioma (2). A cavitated mass was also observed on ultrasonography in muscular angiomatosis (3). Thus, there may be no ultrasonographic pattern that is diagnostic of angiomatosis because of variation in echogenicity and inconsistency in whether the mass echotexture appears heterogeneous or homogeneous. This interpretation is also supported by a previous human case of angiomatosis in which ultrasonography allowed only a provisional diagnosis of hemangioma (35).

In the present case, ultrasonography could not fully demonstrate the anatomical connection between the mass lesions and the adjacent muscular structures, although the histopathological findings indicated that the masses might have originated from or infiltrated the muscle. The limited field of view on ultrasonography may have made this anatomical relationship difficult to identify. When the swollen area of the S1 mass was scanned, disrupted alignment of the muscle fibers was observed in the echotexture of the deep pectoral muscle adjacent to the S1 mass, suggesting intramuscular infiltration of the lesion. This finding partly resembled previous ultrasonographic findings of angiomatosis, in which hypoechoic lesions contained distorted fibers of adjacent muscle (35). However, ultrasonography is limited in its ability to provide significant evidence for detecting the cellular origin of angiomatosis.

The present Doppler ultrasonographic finding of poor vascularity within the mass lesions differed from previous findings showing enriched vasculature within the masses (3, 35). The reason for this difference in mass vascularity between the present and previous ultrasonographic findings remains unclear. However, the severe degenerative and destructive changes identified histopathologically in most vessel-like structures may have contributed to the poor vascularity observed in the present case. Actually, Doppler ultrasonography has previously revealed varying degrees of vascularity within the masses of angiomatosis, as indicated by negative to moderate Doppler signals (24, 25). Variable Doppler signals are also found within hemangiomas, compared to hypervascularity within malignant vascular tumors (25). Thus, it is considered difficult to differentiate angiomatosis from other vascular anomalies, especially hemangioma, using Doppler ultrasonography. Vascular malformations can be subclassified into high-flow malformations, such as arteriovenous vessels, low-flow malformations, such as capillary, cavernous, and venous vessels, and lymphatic malformations, based on the predominant vessel types within the mass (2, 14, 20, 21). Abnormal vasculature within hemangiomas includes capillary malformation in the infantile type and venous and lymphatic malformations in the congenital type (9, 39). Arterial vasculature may also be detected ultrasonographically in these two hemangioma types (39). This intralesional vascularity seemed to differ from the predominant venous vasculature within the mass of angiomatosis (1, 6, 7, 35). Doppler ultrasonography may provide complementary evidence to support the histopathological diagnosis of angiomatosis, if it can detect intralesional arterial or venous vasculature (2, 14, 19, 21–23). Muscular angiomatosis was characterized morphologically by rich vasculature within the affected muscles including a fluid-filled mass (34). Thus, Doppler signals within the affected muscular structure may be the Doppler ultrasonographic finding indicative of muscular angiomatosis.

In the present case, the masses had lower soft tissue attenuation than neighboring structures such as muscle and subcutaneous adipose tissue, which made them easier to distinguish. This CT finding was consistent with previous CT descriptions of angiomatosis, in which the mass showed a well-defined contour because of attenuation differences from neighboring structures (37). However, the variable appearance of the masses themselves has previously been found in the CTs of angiomatosis, showing a heterogeneous structure (37), a cystic structure (31), and intra-mass small tortuous vasculature (28). Additionally, CT of vertebral angiomatosis revealed sponge-like vertebral bone proliferation (27). Thus, no specific CT sign of angiomatosis may make it difficult to differentiate this disease from other vascular tumors and deformations. Lesion size may affect the appearance of soft tissue attenuation, as the largest S1 mass appeared heterogeneous, likely reflecting embedded muscle tissue within the mass. CT has lower soft tissue resolution than magnetic resonance imaging (MRI), although it remains valuable for evaluating calcification and phleboliths, which may occur concurrently with angiomatosis (9, 20, 40). Contrast-enhanced CT can help detect abnormal vascularity and distinguish normal from abnormal structures because of differential contrast enhancement within or around the mass (28). This technique may also enhance the tortuous venous vascular structures typically found within angiomatosis (35, 36). However, contrast-enhanced CT may not always provide a specific sign because contrast resolution in affected soft tissues varies depending on whether the vascular malformation is venous or arterial (21).

CT helps define the spatial relationships of mass lesions to surrounding structures such as bone, muscle, and adipose tissue by reconstructing two-dimensional images in multiple planes (28, 31). CT can visualize pathological relationships between lesions and adjacent structures, including tissue infiltration, destruction, and mass effect, which may cause displacement and deformation (37). Osseous changes can also be evaluated because of the high spatial resolution for bone, such as decreased bone density representing osteolysis, as well as with three-dimensional reconstruction, which was used in the present case to demonstrate rib deformity adjacent to multiple masses in the left thoracic wall (2, 29). Although the exact cause of the mass-associated rib deformity is not fully understood, congenital cutaneous or subcutaneous mass formation may lead to concurrent skeletal deformity in adjacent structures (18, 19). In the present case, the masses occupying the left thoracic wall may have acted as mechanical obstacles that induced rib deformity during development (18, 19). Various vascular lesions in the deep soft tissues can also cause adjacent osseous changes such as bone erosion, deformation, or periosteal reaction (2), these changes have previously been reported in 20% of human patients with congenital hemangiomas (2).

Fine-needle aspiration provided limited cytological evidence for diagnosing juvenile angiomatosis in the present case. The result may be identical to the previous cytological result for the fine needle aspiration specimen, which revealed atypical mesenchymal cells of an unidentifiable origin (33). This technique can frequently disrupt cellular alignment, which is an important feature in the evaluation of malignancy (28, 41). When compared to fine-needle aspiration, Tru-cut biopsy can lead to more indicative histopathological characteristics of this disease, because this technique is effective for obtaining specimens in good condition, in which the cells and their architectural arrangement are preserved (41). However, Tru-cut biopsy allows collection of tissue fragments of a limited size obtained from a mass lesion, resulting in important findings being missed. Thus, wedge biopsy and partial excisional biopsy should be used for the antemortem histopathological evaluation of angiomatosis, as has been done previously (1, 7, 8, 12, 17, 38).

Histopathology can provide definitive evidence for the differential diagnosis of angiomatosis, which is commonly characterized by multiple vascular-like structures contained within the muscle layers (8, 15, 26). The histopathological diagnosis can be strongly supported by immunohistochemistry, allowing the positive immunoreactivity for von Willebrand factor and smooth muscle actin in the present case. This positive immunostaining pattern was the common immunohistochemical finding of angiomatosis (15, 17, 31). Besides combination of these two antibodies, the tissues of angiomatosis were positively immunostained for CD31 and smooth muscle actin (31–33), and CD 31 and von Willebrand factor (8, 26). When considering the further clinical application of histopathological examination, Tru-cut biopsy specimens must need to be used for immunohistochemical examination (42).

MRI is an advanced imaging modality that is effective for diagnosing soft tissue angiomatosis in human medicine (25, 27, 40). Intramuscular infiltrative change, a common concurrent lesion associated with angiomatosis, can be characterized by specific signal intensity patterns (40). The heterogeneous high signal intensity of the affected muscle may represent intramuscular infiltration by a plexus of small venous vessels on T2-weighted fat-saturated images (40). In addition, intramuscular hemangioma may appear as a mixture of hypointense and hyperintense signals on T2-weighted images (20). Thus, MRI might have helped clarify the mass-associated muscular abnormality if it had been used in the present case.

In the present case, ultrasonography and CT scans were valuable for localizing the lesion and assessing anatomical relationships, which were suggestive of muscular involvement in angiomatosis. However, the imaging findings were not pathognomonic, even if the present case could have been examined by MRI. Imaging examinations are just of ancillary significance for histopathological and immunohistochemical evaluations, allowing definitive diagnosis of this disease.

## Conclusion

Ultrasonography and Doppler ultrasonography revealed the parenchymal echotextures of seven masses associated with skin swelling, and poor intra-mass vascularity. CT revealed anatomical relationship among seven masses, and the rib deformity in the areas affected by these masses. However, there was no specific ultrasonographic and CT sign of angiomatosis. The final diagnosis of bovine juvenile angiomatosis accompanied by muscular involvement was made based on the histopathological and immunohistochemical findings showing positive immunoreactivity for von Willebrand factor and smooth muscle actin in vessel-like structures scattered within the muscular structures. The present results highlight the complementary roles of ultrasonography and CT in the assessment of this disease. Definitive diagnosis of this disease can be made by integrating imaging, and histopathological and immunohistochemical examinations.

## Data Availability

The raw data supporting the conclusions of this article will be made available by the authors, without undue reservation.
