# Supplementary material for: Case Report: Diagnosis of multiple juvenile angiomatosis in a newborn calf
Source: Front Vet Sci. 2026 Aug 17;13:1882023. doi: 10.3389/fvets.2026.1882023 (PMC13526633; doi:10.3389/fvets.2026.1882023)
Supplement: Supplementary file 1 [file Data_sheet_1.pdf]

Supplementary Material

Case report: Diagnosis of multiple juvenile angiomatosis in a newborn calf

Takeshi Tsuka*, Kodai Kusuda, Sohta Hishikawa, Natsuki Hashimoto, Yuji Sunden

*** Correspondence:** Takeshi Tsuka: tsuka@tottori-u.ac.jp

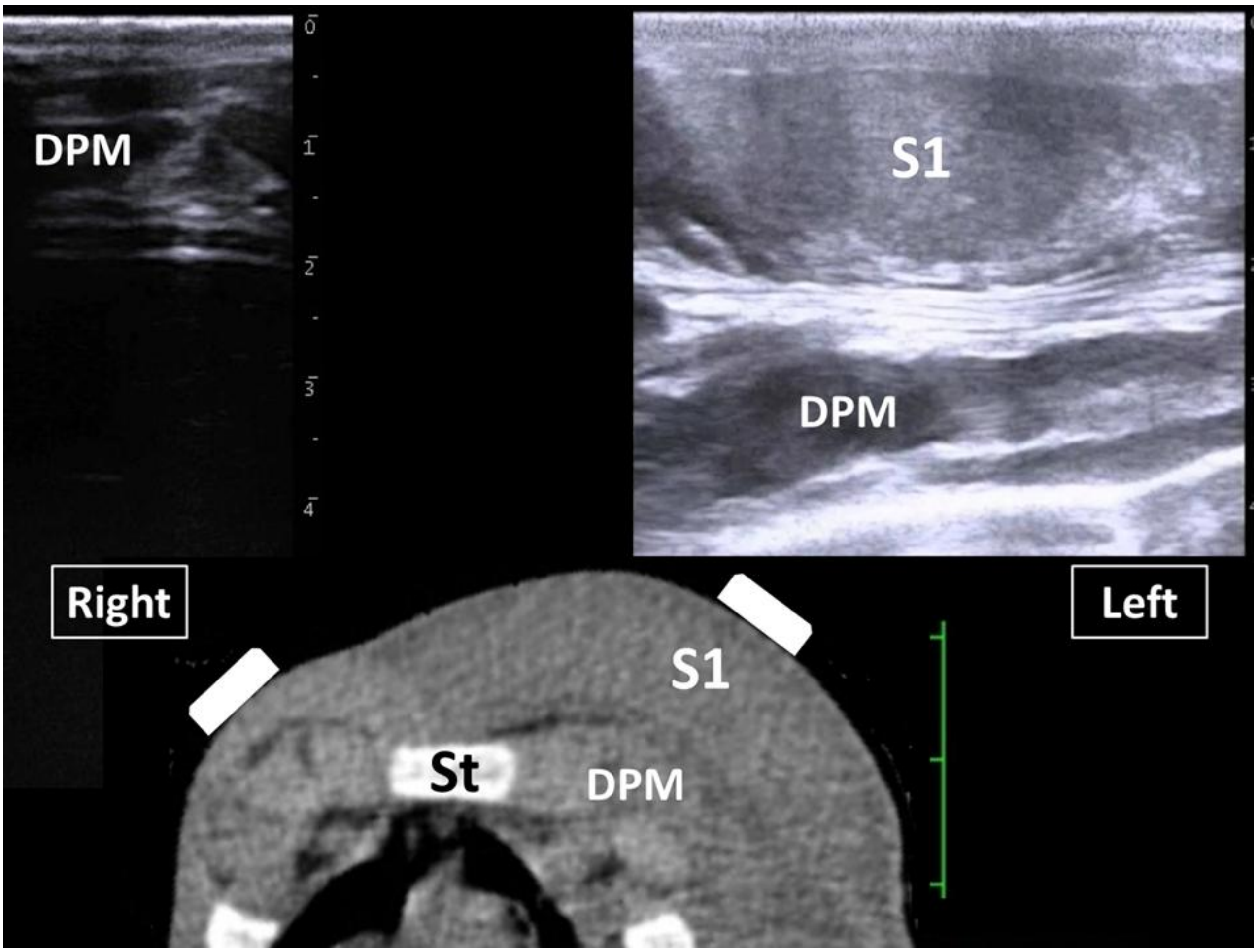

Supplementary Figure 1. Transverse computed tomographic section of the chest showing the transducer positions used to scan the most ventral areas of the left and right thoracic walls. Ultrasonography of the right thoracic wall reveals the normal echotexture of the deep pectoral muscle (DPM), which appears as three homogeneously hypoechoic segments separated from one another.

Compared with the normal DPM, the DPM in the left thoracic wall appears enlarged on the ultrasonographic view, similar to Figure 2A.

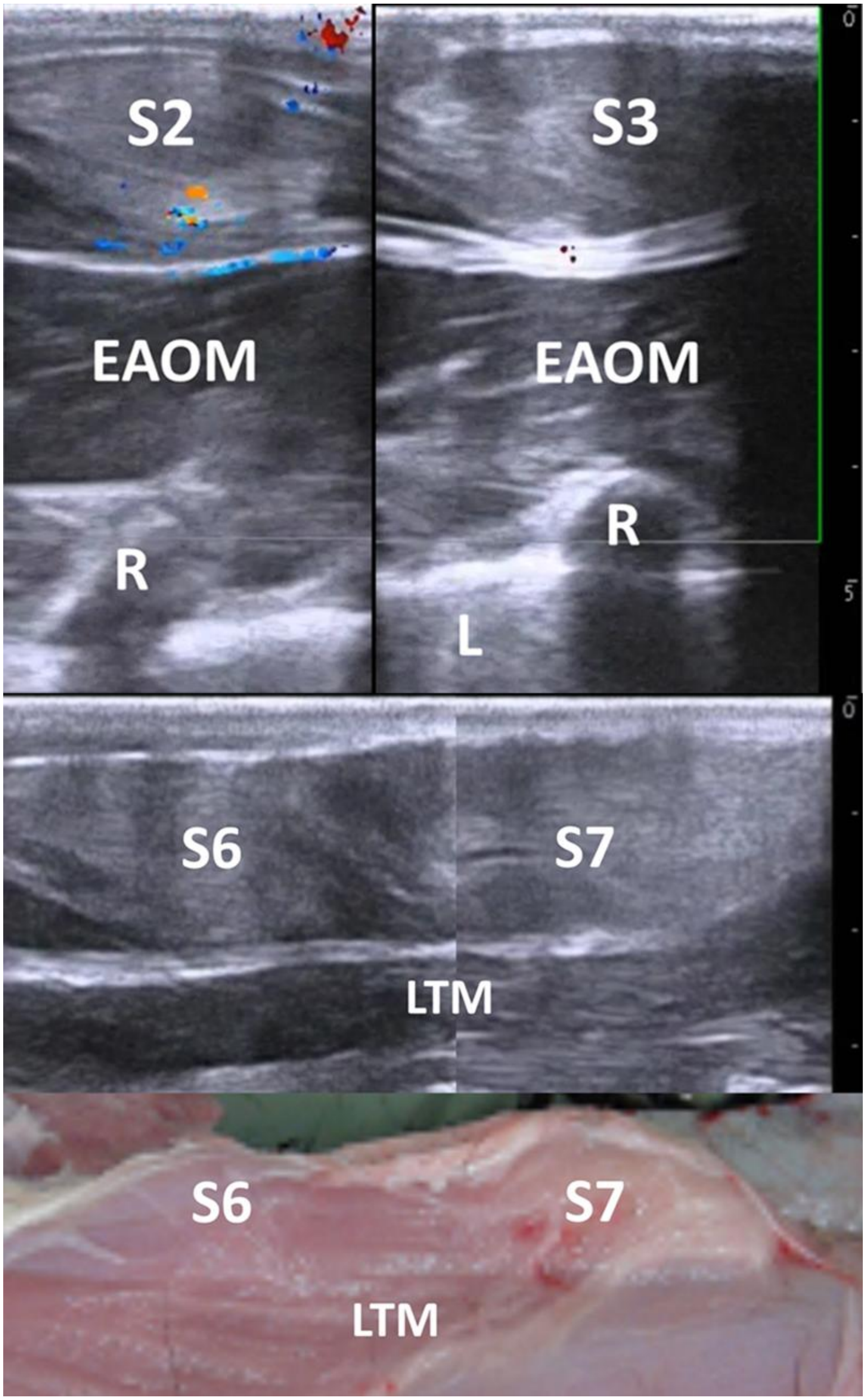

Supplementary Figure 2. The upper left and right panels show transverse ultrasonographic views of the second and third masses (S2 and S3, respectively). In both masses, echogenic oval structures are outlined by hyperechoic membranous structures. The external abdominal oblique muscles (EAOM), which run deeper than the S2 and S3, appear to have normal muscular echotexture. In the areas deeper than both the masses and EAOM, the hyperechoic contour of the ribs (R) with acoustic shadowing and the lung structure (L) are seen. Doppler ultrasonography identifies poor vascularity within the S2 and S3 masses. The middle left and right panels show the longitudinal ultrasonographic views of the sixth and seventh masses (S6 and S7, respectively). These masses run adjacent to the underlying longissimus thoracic muscle (LTM). Both the masses and LTM are seen as separated by the hyperechoic membranous structures. The lower panel includes the macroscopic photo of the S6 and S7 masses corresponding to the ultrasonographic views of the S6 and S7 masses.

Supplementary movie 1. Dorsal computed tomography, corresponding to Figure 3, shows the thorax from the most dorsal to ventral parts. Seven masses are located in the dorsoventral alignment in the left thoracic wall; Sixth and seventh masses (S6 and S7, respectively) at the height of the spinous process in the ninth to thirteenth thoracic vertebrae (T9 to T13) (0 to 6 frames of the movie); Fifth mass (S5) at the level of the sixth to ninth ribs (8 to 14 frames of the movie); Fourth mass (S4) along the seventh to ninth ribs (14 to 24 frames of the movie); Third mass (S3) at the level of the sixth to seventh ribs and more superficial than the S4 mass (18 to 24 frames of the movie); Second mass (S2) at the level of the fifth to seventh, deformed ribs (25 to 34 frames of the movie); and the largest first mass (S1) at the level of the sternum (St) and along the fifth to eleventh, deformed ribs (33 frames to end of the movie).

Supplementary movie 2. Three-dimensional CT of the rib cage shows initially the cranial aspect of the rib cage, followed by the lateral aspect of the right normal rib cage (0 to 22 frames of the movie). Finally, the lateral aspect of the left deformed rib cage is shown, which is characterized by severe rolling toward inside of the thoracic cavity in the sixth to tenth ribs (22 frames to end of the movie). Scale: 50 mm.
